# Supplementary material for: Scale dependence in hydrodynamic regime for jumping on water
Source: Nat Commun. 2023 Mar 17;14:1473. doi: 10.1038/s41467-023-37119-2 (PMC10020434; doi:10.1038/s41467-023-37119-2)
Supplement: Supplementary file 1 — Supplementary Information [file 41467_2023_37119_MOESM1_ESM.pdf]

Supplementary information

Scale dependence in hydrodynamic regime for jumping on water

Minseok Gwon[1], *Dongjin Kim*[1], *Baekgyeom Kim*[1], *Seungyong Han*[1*], *Daeshik Kang*[1*],

and Je-Sung Koh[1*]

[1]Department of Mechanical Engineering, Ajou University, 206 Worldcup-ro, Yeongtong-gu, Suwon-si, Gyeonggi-do, 16499, Republic of Korea

*Address correspondence to:

Prof. Seungyong Han; sy84han@ajou.ac.kr

Prof. Daeshik Kang; dskang@ajou.ac.kr

Prof. Je-Sung Koh; jskoh@ajou.ac.kr

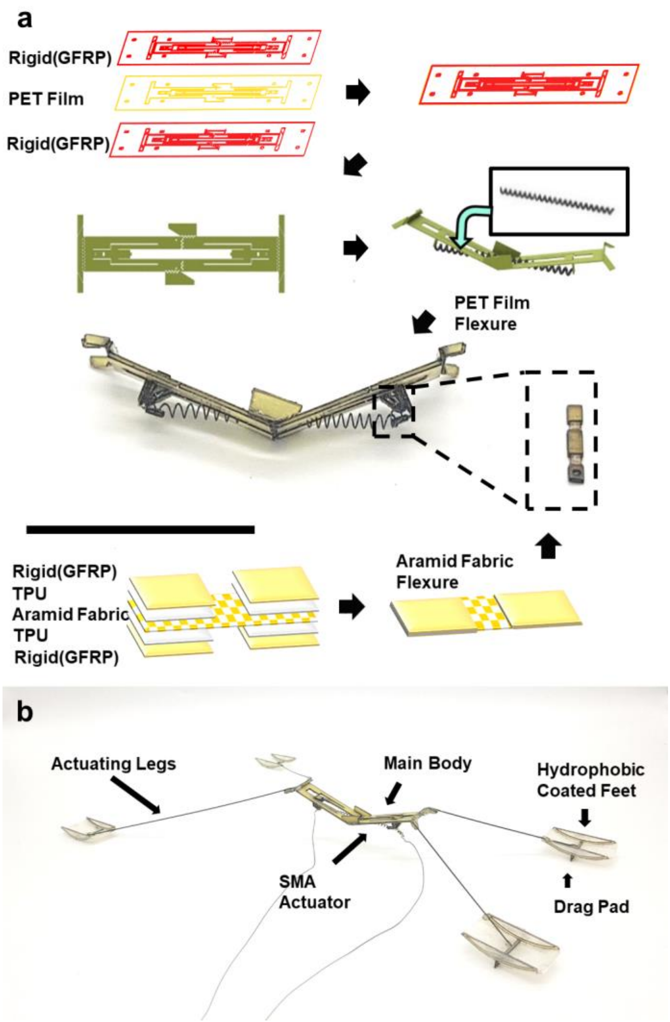

Supplementary Fig. 1. Robot Fabrication. a Design of the robot is based on origami-inspired engineering and made using the composite laminate structure. The composite of the body is made of PET flexure and spring-loaded parts are made of aramid fabric flexure. The robot is driven by a shape memory alloy (SMA) spring actuator. Power was delivered via external wires attached end of the spring actuator. Scale bar, 5 cm. **b** The photograph of the fully-assembled robot.

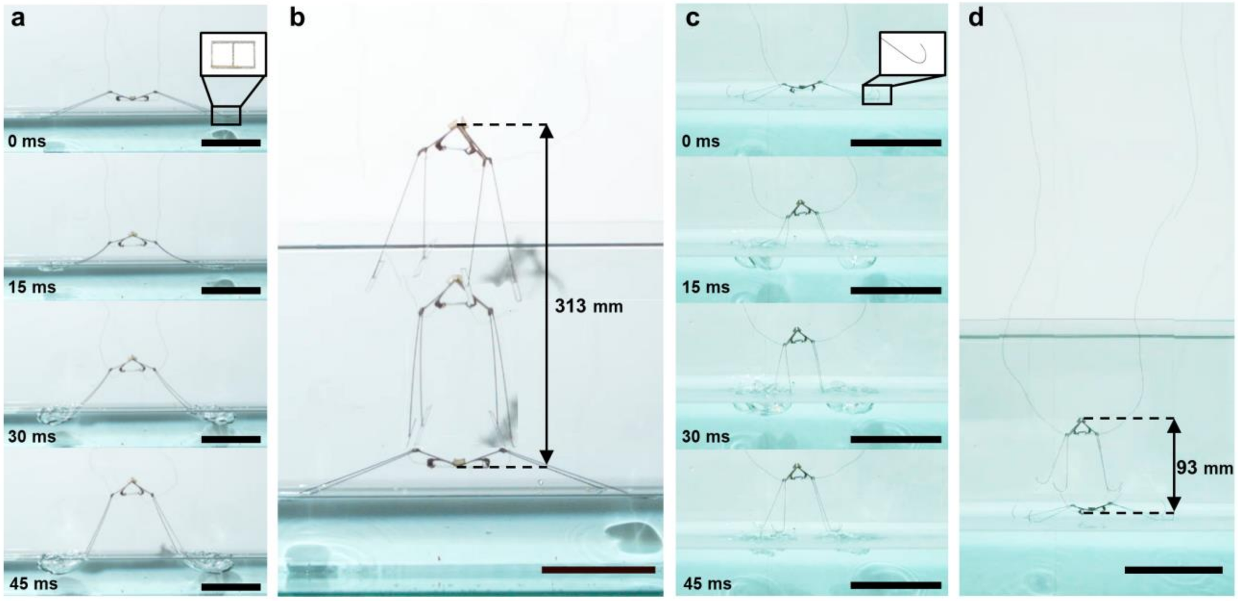

Supplementary Fig. 2. Experimental results of vertical jumping robot B and robot C. a Jumping sequence of robot B with punched rectangular drag pads. Scale bar, 10 cm. **b** Robot B jumps up to 313mm on the water surface. The Weber number of the robot is 34. Scale bar, 10 cm. **c** Jumping sequence of robot C with wire-type legs. **d** The robot C jumps up to 93 mm, and the Weber number of the robot is about 6. Scale bar, 10 cm.

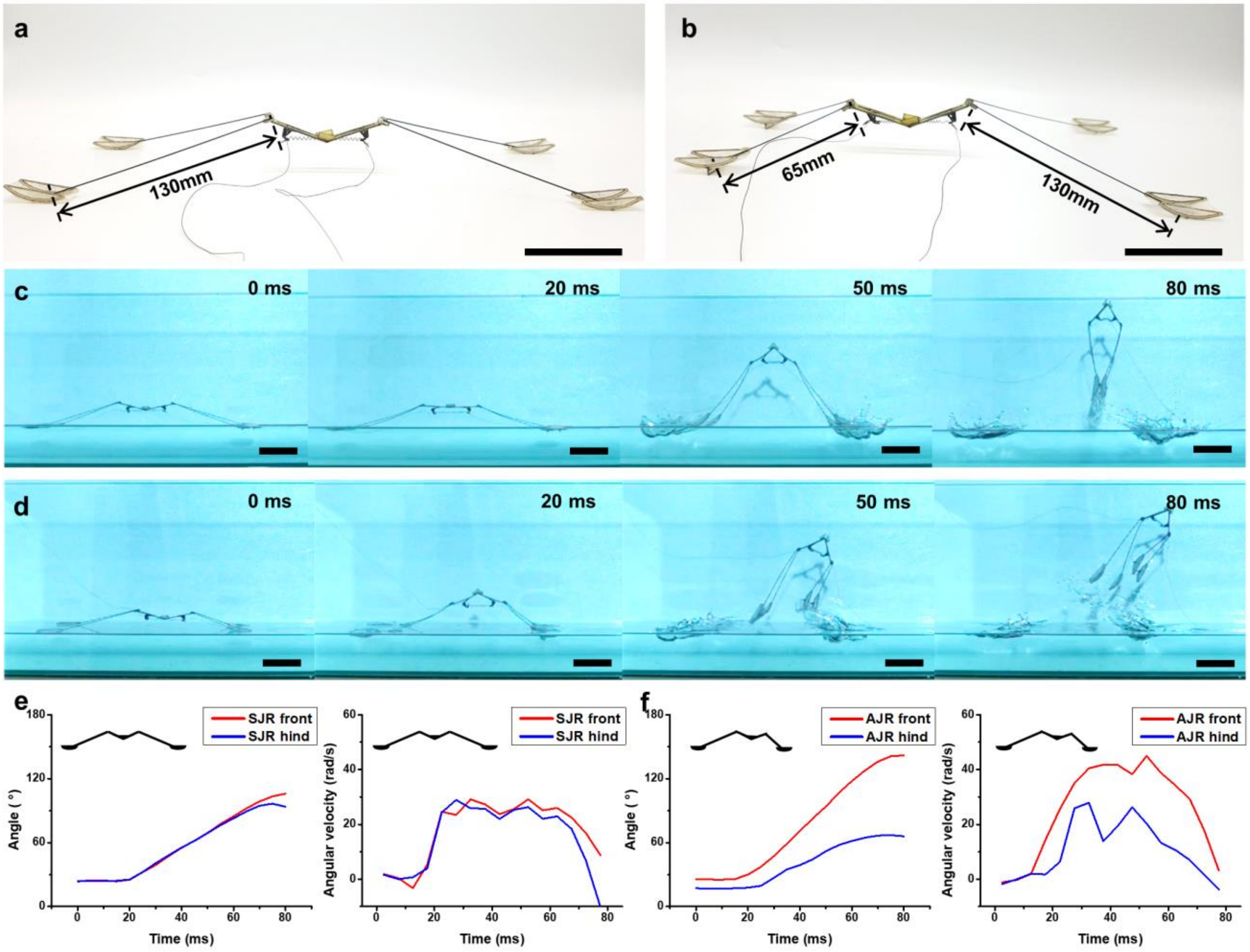

Supplementary Fig. 3. Jumping sequence of SJR and AJR. a Prototype of a SJR with the same leg length of 130 mm. **b** Prototype of an AJR with different leg lengths. The front legs are 65 mm long and the hind legs are 130 mm long. Scale bar, 5 cm. **c, d** Directional jumping sequence of SJR (**c**) and AJR (**d**) attached 2.6 mm drag pads. Scale bar, 5 cm. **e, f** Tracking angle and angular velocity of the SJR (**e**) and AJR (**f**). Red lines indicate the front legs and blue lines indicate the hind legs of each robot. Note that the angular velocity of AJR hind legs is approximately twice that of the hind legs.

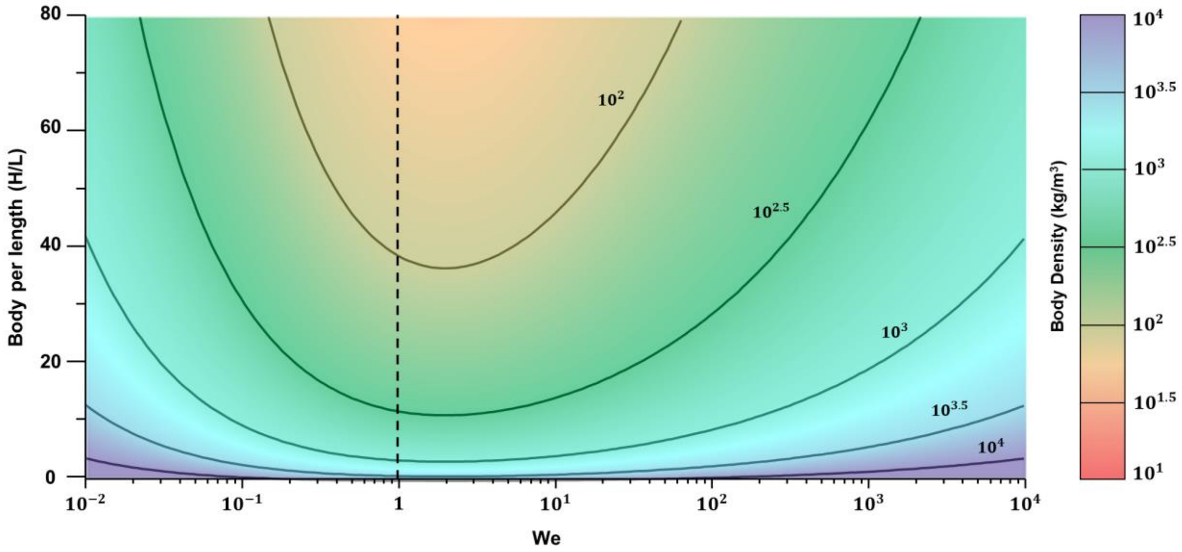

Supplementary Fig. 4. Normalized jumping height related to Weber (We) number. The graph shows a non-linear relationship between jumping height normalized by body length (L) and Weber number. The slope of normalized jumping height is steeper in the surface tension-dominant region than in the drag-dominant region.

Species	Locomotion	L (mm)	M (mg)	V (m/s)	Ba	We	Bo	Re	Source
**Insects**									
Water Strider	Jumping	28	4.59	1.1	0.03	0.06	0.0005	16	[1, 15, 24]
Fisher spider		74*	233	0.6	0.092	1 ~ 10	0.01 ~ 0.1	100 ~ 1000	[3, 13]
Springtail		0.5	0.102	0.63	0.012	0.4	0.0014	50	[4, 15, 26]
Pygmy mole cricket		5.56	9.2	1.3	0.11*	57*	0.0017*	670*	[10]
Basilisk lizards	Running	87*	20800	1.4	22*	670*	20*	24000*	[20, 23, 26]
**Robots**									
Robot 1	Jumping	25*	510	0.09	0.200	4.700	0.0054	260	[13]
Robot 2		140	10200	1.92	1.4*	1000*	5*	66000*	[25]
Robot 3		13	4.08	0.5 ~ 1.0	0.013	7 ~ 28	0.6	1000	[15]
Robot 4		95*	68	1.67	0.060	0.165	0.001	48	[9]
**This study**									
Robot A	Jumping	320	3000	3.6	1.021	131	1.225	3560	
Robot B		320	3000	2.46	0.427	34.340	0.081	3560	
Robot C		160	680	1	0.075	6.050	0.0049	134.1	

Supplementary Table 1. Data sheet of the animals and robots jumping on water. L represents body length (mm) including the leg length, *M* represents mass (mg), *V* is jumping velocity (m/s), Ba is Baudoin number, We is Weber number, Bo is Bond number, and Re is Reynolds number. Values with * symbols in the blue are approximated values calculated from videos and images in the literature.

Design type	Height of lateral drag pads	Mass (g)	Body length (mm)	Jumping Velocity (m/s)	Jumping angle (°)	Jumping height (mm)	Travel length (mm)
SJR 1	X	3±0.1	320	3.57	88.2	545	32
SJR 2	2.1 mm			3.39	85.4	446	298
SJR 3	2.6 mm			3.11	75.5	376	425
SJR 4	3.6 mm			2.99	63.4	313	498
AJR 1	X	3±0.1	280	3.43	84.3	526	62
AJR 2	2.1 mm			3.11	74.5	396	482
AJR 3	2.6 mm			3.02	64.2	316	556
AJR 4	3.6 mm			2.83	49.4	237	539

Supplementary Table 2. Data sheet of the SJRs and AJRs jumping on water.
