# Supplementary material for: Scale dependence in hydrodynamic regime for jumping on water
Source: Nat Commun. 2023 Mar 17;14:1473. doi: 10.1038/s41467-023-37119-2 (PMC10020434; doi:10.1038/s41467-023-37119-2)
Supplement: Supplementary file 3 — Description of Additional Supplementary Files [file 41467_2023_37119_MOESM3_ESM.pdf]

Description of Additional Supplementary Files

Title: Supplementary Movie 1

Description: Vertical, directional, and obstacle jump on the water surface.

This video shows the jumping robots designed with a high Weber number (We = 131) overcoming the obstacle (height = 220 mm), jumping up to 545 mm in the vertical direction, and jumping forward 556 mm in the lateral direction.

Title: Supplementary Movie 2

Description: Comparison of jumping robots with various Weber numbers

This video shows the difference in jumping performance and sequences during take-off of jumping robots with different Weber numbers. The robots are designed to have a specific range of Weber numbers (1<We<10, 10<We<100, 100<We) through the shape of the legs and the size of the robots.
